# Case Report and Literature Review of Occupational Transmission of Monkeypox Virus to Healthcare Workers, South Korea

**DOI:** 10.3201/eid2905.230028

**Published:** 2023-05

**Authors:** Yunsang Choi, Eun-bi Jeon, Taeyoung Kim, Seong Jin Choi, Song Mi Moon, Kyoung-Ho Song, Hong Bin Kim, Eu Suk Kim

**Affiliations:** Seoul National University Bundang Hospital, Seongnam, South Korea (Y. Choi, E.-b. Jeon, S.J. Choi, S.M. Moon, K.-H. Song, H.B. Kim, E.S. Kim);; Seoul National University College of Medicine, Seoul, South Korea (Y. Choi, S.J. Choi, S.M. Moon, K.-H. Song, H.B. Kim, E.S. Kim);; Korea Disease Control and Prevention Agency, Cheongju, South Korea (T. Kim)

**Keywords:** monkeypox virus, mpox, viruses, zoonoses, healthcare, healthcare worker patient transmission, occupational injuries, needlestick injuries, South Korea

## Abstract

We report a case of occupational monkeypox virus infection from a needlestick injury in a healthcare worker in South Korea and review similar reports in the literature during 2022. Postexposure prophylactic treatment with a third-generation smallpox vaccine and antiviral agent tecovirimat inhibited local virus spread and alleviated lesion pain.

In July 2022, the World Health Organization (WHO) declared the international mpox outbreak a global public health emergency (*1*). Mpox, caused by monkeypox virus, is transmitted through person-to-person contact, contaminated objects, or respiratory droplets (*2*). During the outbreak, transmission occurred through sexual contact in most reported mpox cases, especially among men who have sex with men (*3*).

By December 2022, the WHO had reported 83,497 confirmed cases of mpox, including 1,176 cases among healthcare workers (HCWs). However, most infections of HCWs occurred in the community, rather than from occupational exposure. Further investigations are needed to determine the main risk of occupational exposure to monkeypox virus in hospitals and the best responses for prevention (*4*).

Cases of HCWs who were confirmed to have monkeypox virus infections obtained through needlestick injuries or contact with a contaminated environment while collecting patient specimens have been reported recently in several countries (*5*–*10*). The first imported case of mpox was reported in South Korea in June 2022 (*11*). We report a case of occupational monkeypox virus transmission in an HCW in Korea. Furthermore, we conducted a literature review of other reported cases of healthcare-associated monkeypox virus transmission in 2022.

## The Study

On November 14, 2022, a 33-year-old healthy female HCW was exposed to monkeypox virus through a needlestick (26G needle) injury on the left index finger during aspiration of an infected patient’s vesicle. The HCW wore personal protective equipment consisting of a disposable gown, double gloves, and powered air-purifying respirator. Bleeding occurred after the injury, and povidone-iodine was applied to the puncture site ≈20 min after the incident. Within 20 hours after injury, the HCW received a third-generation smallpox vaccine (single-dose, subcutaneous injection of JYNNEOS; Bavarian Nordic A/S, https://www.bavarian-nordic.com) as postexposure prophylaxis. The HCW had no history of smallpox vaccination, exposure to monkeypox virus, or recent sexual contact and had not traveled abroad.

On November 17 (day 1), the HCW noticed a small papule at the needlestick site (Figure, panel A). On day 2, the papule increased in size; the HCW was admitted to the hospital isolation ward, according to Korea Disease Control and Prevention Agency policy for mpox. We performed monkeypox virus-specific PCR on blood samples and oropharynx and nasopharynx swab samples collected from the HCW. All samples were PCR-negative for monkeypox virus.

**Figure:**
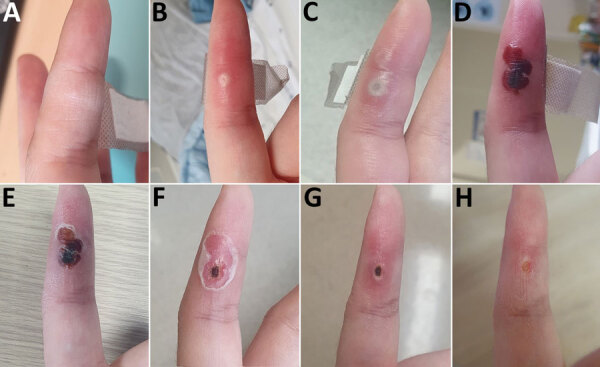
Progression of skin lesion caused by occupational transmission of monkeypox virus to a healthcare worker in South Korea. The healthcare worker was infected in the left index finger with monkeypox virus after a needlestick from a 26G needle during aspiration of an infected patient’s vesicle. Photographs show the lesion at the inoculation site from onset to recovery. A) Day 1 (November 17, 2022); B) day 6; C) day 8; d) day 18; E) day 22; F) day 25; G) day 34; H) day 40.

On day 6, the lesion was slightly larger and had a central umbilication (Figure, panel B). We aspirated the lesion, and PCR results for the aspirate were positive for monkeypox virus. We repeated testing of blood samples and oropharyngeal and nasopharyngeal swab samples, and PCR results for those samples were negative. Monkeypox virus transmission was presumed to be occupational because no other risk factors were identified. 

On day 8, a new lesion appeared immediately above the initial lesion and began to progress (Figure, panel C). Pain at the lesion site was severe; the numeric rating scale (*12*) score was 8 because of neuralgia. The HCW described a sharp pain as “the feeling of being cut with a knife” that disrupted sleep. Although no disseminated lesions were present, because of the pain intensity and local spread of infection around the initial lesion, the attending infectious disease specialist prescribed tecovirimat starting on day 9 (10 days after smallpox vaccination), which substantially alleviated the pain. By day 16, the pain was almost completely gone.

On day 18, the lesions formed a crust (Figure, panel D) and were partially debrided 4 days later (Figure, panel E). PCR of the debrided skin specimen was positive for monkeypox virus. On day 25, the crust was completely debrided. and a necrotic scab remained underneath the devitalized tissue at the puncture site (Figure, panel F). Because mpox lesions developed after postexposure vaccination, the HCW did not receive a second dose of smallpox vaccine, which was scheduled for 28 days after the first dose. PCR of a lesion site sample yielded positive results for monkeypox virus, but the possibility of virus transmission was low, and clinical progress was stable. Consequently, we discharged the HCW under the guidance of an infectious disease specialist. After the patient was discharged, the tissue around the puncture site recovered completely by day 34 (Figure, panel G), and the scab was completely eliminated by day 40 (Figure, panel H).

We conducted a literature review to evaluate the status of and response to monkeypox virus infections among HCWs during the 2022 outbreak (Table). Transmission of monkeypox virus occurred through needlestick injuries in 5/8 cases; initial lesions developed at the puncture sites in each of those cases. The median incubation period was 5 (range 3–10) days, which was slightly shorter than the previously reported 7- (range 3–20-) day incubation period (*3*). The patient we report did not have disseminated or severe mpox. However, after administration of tecovirimat, symptoms (especially pain intensity) improved substantially and rapidly.

**Table:** Case report of occupational transmission of monkeypox virus to a healthcare worker in South Korea and literature review of healthcare-associated cases of monkeypox virus transmission during the 2022 mpox outbreak*

Characteristics	Case reports							
	1	2	3	4	5†	6†	7‡	8
Country	France	Brazil	Portugal	Florida, USA	Brazil	Brazil	California, USA	South Korea
Exposure date, 2022	July	July	July	July	July	July	August	November
HCW, age/sex	25/F	20s/F	29/M	NA	NA/F	NA/F	40/F	33/F
HCW occupation	Doctor	Nurse	Doctor	Nurse	Nurse	Nurse	Doctor	Doctor
Patient source, age/sex	30/M	20s/M	NA	NA	40/M	40/M	NA	20s/F
Exposure								
Type	Needlestick	Needlestick	Needlestick	Needlestick	Fomites	Fomites	Other§	Needlestick
Site	Right thumb¶	Thumb	Left index finger	Index finger	Left ring finger	Forearm	Left middle finger	Left index finger#
PCR results								
Vesicle	Positive	Positive	Positive	Positive	Positive	Positive	Positive	Positive
Oropharynx	Negative	Positive	Negative	NA	NA	NA	NA	Negative
Blood	Negative	Positive	Negative	NA	NA	NA	NA	Negative
Incubation period, d	4	5	4	10	5	5	7	3
Dissemination	No	Yes	Yes	No	Yes	Yes	Yes	No
Vaccination**	Imvanex, <3 h	No	No	JYNNEOS, <15 h	No	No	No	JYNNEOS,<20 h
Isolation of HCW after symptoms, d	21	19	24	19	21	22	20	26
Tecovirimat treatment	No	No	No	No	No	No	Yes, 14 d	Yes, 14 d
Major symptoms								
Skin lesion(s)	Yes	Yes	Yes	Yes	Yes	Yes	Yes	Yes
Lymphadenopathy	No	Yes	Yes	No	Yes	Yes	No	No
Other	Seropurulent fluid from wound	Fever	Fever, chills, myalgia	No	Hyperemia	Fever	Fever, cough, sore throat	Myalgia
Reference	(*5*)	(*6*)	(*7*)	(*8*)	(*9*)	(*9*)	(*10*)	This case

*Cases 1–7 were collected from the literature; case 8 is the patient described in this report. HCW, healthcare worker; NA, not available. †Cases 5 and 6 were described in 1 report. ‡Medical history of rheumatoid arthritis, treated with etanercept. §Inadvertent contamination during specimen collection or contact with contaminated environment. ¶Puncture from 25G needle. #Puncture from 26G needle. **Postexposure prophylaxis vaccination with third-generation smallpox vaccines (JYNNEOS, also known as Imvanex; Bavarian Nordic A/S, https://www.bavarian-nordic.com) conducted within specified hours after exposure to monkeypox virus.

## Conclusions

As recommended by WHO (*1*), 3 HCWs with needlestick injuries, 2 from the literature (*5*,*8*) and the HCW in the case we report, received a third-generation smallpox vaccine promptly after needlestick injury, and only local skin lesions developed at the site of inoculation without generalized illness. However, additional reports from the literature showed that HCWs without postexposure vaccination had substantially disseminated lesions; among those, 2 HCWs (*6*,*7*) were infected by needlestick injuries. Lesions developed on the hands and wrists of the other 2 HCWs, and the mode of transmission was likely fomite contact with bare skin. The HCW from California (*10*) was immunocompromised and worked in a clinic where patients with mpox regularly visited; unrecognized exposure and spread might have occurred through respiratory droplets. 

On the basis of our case report and literature review, we recommend the following procedures for HCWs who treat patients with mpox. First, the literature review revealed differences in clinical manifestations depending on the infection route and vaccination status, similar to findings from previous reports from the prairie dog–associated mpox outbreak in the United States (*13*). Therefore, prompt vaccination after exposure might prevent disease progression and should be considered for HCWs in environments requiring contact with monkeypox virus-infected patients; preexposure vaccination should also be considered. Second, precautions should be exercised when collecting specimens from patients with suspected mpox. For the safety of HCWs, instead of unroofing or aspirating the lesion with a sharp tool, the sample should be obtained by rubbing the surface of the lesion with a swab or collecting a scab with forceps (*14*). Because PCR is highly sensitive, a positive result can be obtained when samples are collected by using this method. Third, although tecovirimat is generally recommended for patients with severe mpox or high risk of dissemination (*10*), the drug was administered to our patient, who had localized infection, to prevent disease progression; prompt administration of tecovirimat might be necessary to maximize effectiveness. Most patients with mpox report extreme pain in the affected area. Thus, although the isolation period or the time until the virus is undetectable might not be shortened, antiviral treatment should be considered if skin lesions progress or pain is severe and no shortage of drugs exists.

In summary, we report a case of monkeypox virus infection in a HCW after a needlestick injury and a literature review of similar cases during the 2022 mpox outbreak. Although larger studies are needed to determine efficacy of postexposure vaccination prophylaxis, this case series indicates postexposure vaccination might have prevented dissemination of virus lesions. Therefore, clinicians should consider postexposure vaccination and tecovirimat or other antiviral drugs to inhibit local monkeypox virus spread and alleviate lesion pain.
